# Implementation and modification of an organizational-level intervention: a prospective analysis

**DOI:** 10.1186/s43058-022-00296-0

**Published:** 2022-06-03

**Authors:** Elisa J. Gordon, Jefferson J. Uriarte, Naomi Anderson, Elida Romo, Juan Carlos Caicedo, Michelle Shumate

**Affiliations:** 1 Department of Surgery - Division of Transplantation, Center for Health Services and Outcomes Research, Center for Bioethics and Medical Humanities, Feinberg School of Medicine, Northwestern University, 633 N. St. Clair, 20th Floor, Chicago, IL 60611 USA; 2 Center for Health Services and Outcomes Research, Feinberg School of Medicine, Northwestern University, Chicago, IL USA; 3 School of Education and Policy, Northwestern University, Evanston, IL USA; 4 Department of Neurology – Biological Sciences Division, University of Chicago, Chicago, IL USA; 5 Department of Surgery - Division of Transplantation, Feinberg School of Medicine, Northwestern University, Chicago, IL USA; 6 Department of Communication Studies, Northwestern University, Evanston, IL USA

**Keywords:** End-stage kidney disease, Health disparities, Organizational culture, Hybrid study, Hospital-based intervention, Equity, Organizational-level intervention, Complex interventions, Culturally competent care

## Abstract

**Background:**

Modifications to interventions can jeopardize intervention outcomes. Pre-existing perceived barriers and facilitators to the intervention arising in the implementation preparation phase may help explain why modifications to the intervention may occur during the implementation phase. This two-site comparative case study describes modifications made to a complex organizational-level intervention and examines how known implementation science factors may have enabled such changes to occur.

**Methods:**

Northwestern Medicine’s^TM^ Hispanic Kidney Transplant Program (HKTP) is a culturally competent transplant center-based intervention designed to reduce disparities in living donor kidney transplantation among Hispanics. In-depth qualitative interviews and discussions were longitudinally conducted with transplant stakeholders (i.e., physicians, administrators, clinicians) at two kidney transplant programs with large Hispanic populations during implementation preparation and implementation phases. The Consolidated Framework for Implementation Research (CFIR) guided interview design and qualitative analysis, and Stirman’s Framework for Reporting Adaptations and Modifications-Expanded (FRAME) guided modification classification.

**Results:**

Across sites, 57 stakeholders participated in an interview, group discussion, and/or learning collaborative discussion. Site-B made more modifications than Site-A (*n* = 29 versus *n* = 18). Sites differed in the proportions of delaying/skipping (Site-A 50% versus Site-B 28%) and adding (Site-A 11% versus Site-B 28%) but had comparable substituting (Site-A 17% versus Site-B 17%) and tweaking (Site-A 17% versus Site-B 14%) modification types. Across sites, the transplant team consistently initiated the most modifications (Site-A 66%; Site-B 62%). While individuals initiated slightly more modifications at Site-B (21% versus Site-A 17%), institutions instigated proportionately slightly more modifications at Site-A (17% versus Site-B 10%). CFIR inner setting factors (i.e., structural characteristics, culture, available resources, implementation climate) that prominently emerged during the implementation preparation phase explained similarities and differences in sites’ modification numbers, types, and agents in the implementation phase.

**Conclusion:**

Organizations implementing a culturally competent care intervention made modifications. CFIR inner setting factors emerging in the implementation preparation phase largely explained similarities and differences in study sites’ modifications. Identifying factors contributing to modifications may help institutions become better prepared to implement an intervention by addressing known factors in advance, which may foster greater fidelity leading to desired outcomes.

**Trial registration:**

ClinicalTrials.govNCT03276390. We registered the study retrospectively on 9-7-17.

**Supplementary Information:**

The online version contains supplementary material available at 10.1186/s43058-022-00296-0.

**Contributions to the literature**

- Research has shown that the CFIR explains why organizations modify interventions. However, research has seldom focused on complex, multilevel interventions with multiple components and strategies and has not examined the nature of those modifications.
- Applying Stirman’s FRAME typology of modifications to the study of implementing a complex intervention (e.g., requiring multiple, interdependent changes in multiple departments), we found that some CFIR elements are associated with different types of modifications.
- These findings demonstrate the groups of CFIR factors that influence modifications, an essential implementation outcome.

## Background

Translating interventions into practice is challenging because intervention components may not fit within unique organizational contexts. The modifications made to better fit the intervention with the context can undermine intervention fidelity [1]. Implementation fidelity, the extent to which intervention is carried out as intended, is essential for evidence-based interventions to achieve their desired outcomes [2]. Determining an acceptable amount and type of intervention modifications remains a challenge of balancing fidelity with ensuring that interventions are implemented with the best fit [3].

Following Stirman et al., we define “modification” as planned or unintentional “changes to the design or delivery of an intervention.” [4] We distinguish modifications from adaptations, which are deliberately made before initiating intervention implementation in new settings or populations [5, 6]. Modifications can be classified in terms of who makes them, what intervention component is modified, the nature of the modification, and the reason for the modification [4, 7]. Examining modifications is important for understanding the potential for positive and negative effects on desired outcomes [8]. Modifications may be harmful or helpful to achieving the intervention’s outcomes, depending on whether intervention components are deemed necessary or proscribed [4, 8]. Assessing the types and levels of modifications can offer greater insights than fidelity monitoring alone. Such examination can demonstrate how modifications affect desired intervention outcomes and illuminate ways to enhance intervention fit within specific settings [4].

Relatively little is known about modifications made during intervention implementation. Studies have examined modifications by culturally adapting prevention interventions to improve fit with clients and optimize desired outcomes [6, 9–11]. The most common types of cultural adaptations are tailoring and adding elements [12]. Systematic reviews and other research on evidence-based interventions targeting the individual provider, staff coordination, and patient levels found that most modifications involved tailoring/tweaking/refining and adding components and were instigated by individual practitioners [4, 12]. One study found that changes or deletions were the most common modifications to a community-based intervention [13].

Identifying determinants of modifications may reveal which intervention components worked in achieving desired outcomes, the circumstances by which they worked [14], and help to prevent modifications from occurring in future research. Stirman’s Framework for Reporting Adaptations and Modifications-Expanded (FRAME) identifies reasons for modifications, which include both the intent of modifying (e.g., increase reach, improve feasibility, reduce costs) and contextual factors driving the decision to make the modifications (e.g., sociopolitical, organizational, provider, and recipient) [7].

However, little research has examined how organizational factors, identified by FRAME or other implementation science theories and frameworks, explain intervention modifications. In one of the few exceptions, Lau et al. examined therapist and practice-level factors that predict different adaptation types. The researchers examined whether the practice influenced augmenting or reducing/reordering adaptations but did not examine the features of those practices that explain differences [15]. Recently, an outcomes addendum was added to the Consolidated Framework for Implementation Research (CFIR) to account for both implementation and intervention outcomes [16]. Only a few studies have previously used the CFIR to examine outcomes [16]. For example, Hasson et al. evaluated factors affecting the fidelity of a complex care intervention for frail elderly involving collaboration between an emergency department nurse, hospital ward staff, and a multi-professional team [17]. Using CFIR, they found that high staff enthusiasm for the project and team responsiveness prompted many additions. Contextual factors, like the lack of financial resources, resulted in less staff attending care planning meetings, while staff with prior positive experiences in similar projects increased enthusiasm for the project [17].

While prior research on intervention modifications has focused primarily on simple interventions that intervene at the patient or physician level, few studies have examined modifications occurring at the organizational level [4]. Healthcare organizational-level interventions are fundamentally complex because they typically involve multiple components at the patient, provider, micro-system (healthcare team), and system levels [18–20]. US hospitals vary (e.g., hospital systems, hospital networks, academic, community) [21] and maintain different organizational structures, cultures, and priorities. Consequently, modifications made to complex organizational interventions raise uncertainty over whether intervention effects can be generalizable to other organizational settings [22]. Scholars have therefore called for a broader understanding of modifications made to “fit provider characteristics, organizational contexts, and service settings (e.g., historical, political, and economic contexts)” [23].

Although recently proposed by Damschoder and colleagues, studies have not yet evaluated how CFIR factors, identified in the preparation phase, engender modifications during the implementation phase [16]. Understanding perceived barriers and facilitators to the intervention arising in the pre-implementation period may explain why certain types of intervention modifications arise during the implementation process, as recommended [14]. A prospective, longitudinal assessment of modifications before implementing interventions can illuminate contextual precursor factors that may enable modifications to occur [14].

### Objectives

This research is part of a large, multi-part 5-year study examining the implementation and effectiveness of Northwestern Medicine’s^TM^ Hispanic Kidney Transplant Program. This paper uniquely examines the types and characteristics of modifications made to a complex, organizational-level, culturally competent care intervention. It describes how implementation science factors in the implementation preparation phase may have enabled modifications to occur during the first year of the implementation phase.

## Methods

### Frameworks

The CFIR [24, 25] was used to describe the contextual factors contributing to modifications and generate comparative site descriptions [26]. CFIR synthesizes myriad implementation science frameworks designed to understand and explain factors influencing implementation outcomes. CFIR comprises five domains: Intervention characteristics, Outer setting, Inner setting, Characteristics of individuals, and Process. Subsequently, the CFIR addendum conceptualized outcomes for use with the CFIR. This study focuses on the connection between CFIR factors and implementation outcomes. Implementation outcomes describe “the success or failure of the implementation” (p. 3) [16]. However, rather than examining implementation success, we take a more nuanced tact, examining modification as an implementation outcome.

Stirman’s FRAME was used to classify and determine the intent of modifications [4]. The FRAME is a coding system designed to classify intervention modifications to understand how modifications influence intervention implementation. The FRAME consists of eight categories: (1) when in the implementation process the adaptation occurred, (2) whether the adaptation was planned or unplanned, (3) who participated in the decision to modify, (4) what is modified, (5) level of delivery the adaptation was made, (6) the type or nature of context or content-level modifications (e.g., tweaking, adding, skipping), (7) whether the adaptation was fidelity consistent, and (8) the reasons for the adaptation, including (a) the goal or intent of the adaptation and (b) contextual factors that influenced the adaptation. We adapted the categories within the “Who participated in the decision to modify” to the HKTP intervention, as Stirman and colleagues recommended [7], by reclassifying the categories as “individual” (i.e., one stakeholder), “transplant team,” and “institution” (i.e., institutional leadership, organizational policies). However, we did not include Stirman’s categories “level of delivery the adaptation is made”, "training and evaluation", and subcategory “contextual factors that influenced the adaptation” because they did not apply to the intervention and CFIR covered contextual factors, respectively. Although the FRAME’s contextual factors overlap with CFIR constructs, we used CFIR because it accounts for a broader array of reasons for modifications that are not addressed by FRAME, including *structural characteristics*, *culture*, *self-efficacy*, and *compatibility*.

### Overall study design

We conducted a type-2 hybrid effectiveness-implementation study design with a pre-post intervention evaluation to assess the impact of the HKTP at two intervention sites (Site-A and Site-B) and two matched control sites. The pre-implementation period spanned from January 1, 2011 to December 31, 2016. The intervention period spanned from January 1, 2017 to March 15, 2020, and ended prematurely due to the COVID-19 pandemic. Our previous research [27] evaluated the impact of the HKTP intervention, including fidelity, on the likelihood of receiving a living donor kidney transplant. The study used an implementation-effectiveness hybrid design and included 2063 recipients. Site-A exhibited greater fidelity to the intervention than Site-B. The HKTP improved the LDKT rates for Hispanic patients at Site-A by 47%. The intervention had no effect at Site-B. Additional findings are described elsewhere [27].

### The HKTP intervention

Northwestern Medicine’s™ Hispanic Kidney Transplant Program (HKTP) is a complex healthcare organizational intervention [26] designed to increase live donor kidney transplant (LDKT) rates among Hispanics. The disproportionately lower LDKT rates by Hispanics and other minority groups are attributed to recipient donor, healthcare provider, and health system factors [28]. The HKTP addresses these factors, including Hispanics’ lack of kidney transplant knowledge, cultural beliefs about transplantation, shortage of bilingual staff, and lack of culturally competent care [29–31]. An additional file describes the HKTP components in detail [see Additional file 1]. A detailed description of procedures, materials, and processes used in the intervention can be found elsewhere [26, 32, 33].

### Present study design

We extend this previous work [27] by conducting a comparative case study to examine the influence of CFIR pre-implementation factors on the types and characteristics of modifications made to a complex organization-level intervention during the first year of implementation. We assessed stakeholders’ shared perceptions of barriers and facilitators to the HKTP pre-implementation period by highlighting how stakeholders made sense of the intervention and their roles in implementing it within their broader organizational cultural context [34]. Qualitative data were valuable for generating in-depth insights into intervention implementation and modification characterization [34]. We used the Template for Intervention Description and Replication [35] and the Consolidated Criteria for Reporting Qualitative research for quality reporting [36]. Northwestern University’s Institutional Review Board granted study approval (STU00201331). Written and verbal informed consent was obtained from individuals participating in one-on-one interviews and group discussions, respectively.

### Setting and participants

The HKTP intervention was implemented at two U.S. kidney transplant programs: in the South (Site-A) and the Southwest (Site-B). Sites were chosen because they perform fifty or more LDKTs per year, have a Hispanic bilingual transplant physician, and serve a large Hispanic patient population. Both were non-profit hospitals with LDKT disparities for Hispanics, as compared to non-Hispanic Whites in 2016 [32]. Site-A was a regionally based, academic affiliated medical center with a large-sized 1000-bed hospital and a level 1 trauma center. Site-B was part of a national academic medical center with a medium-sized 300-bed hospital and no trauma center. The implementation preparation phase occurred between April and December 2016 to prepare for a January 2017 launch. Eligible participants included transplant stakeholders involved in HKTP implementation: physicians (e.g., surgeons, nephrologists, urologists), administrators, clinical staff (e.g., nurses, social workers, schedulers), and others (e.g., IT, marketing, research staff). The study co-Principal Investigators (co-PIs) requested that stakeholders involved in HKTP implementation participate in one-on-one interviews and group discussions via email, phone, and/or in-person, in advance of site visits. Stakeholders were aware of study goals and knew they would engage with the study co-PI through research activities. Participants did not have a relationship with the interviewer (co-PI, EJG) prior to study commencement.

### Implementation strategies

**Fig. 1:**
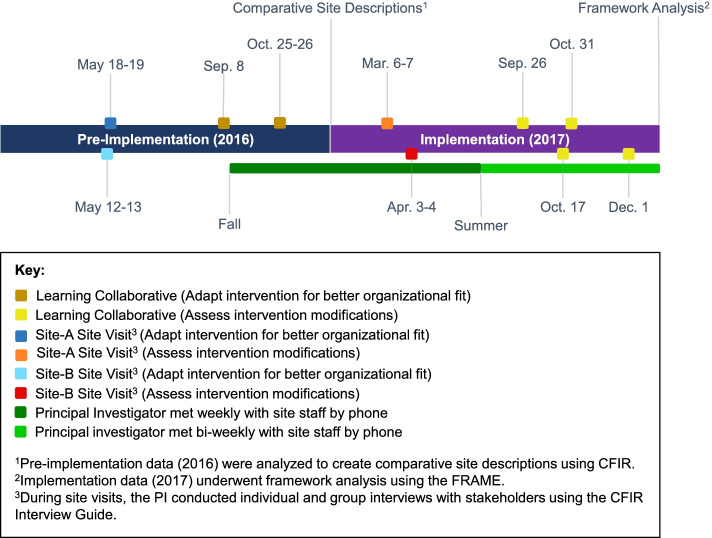
Data collection timeline

The CFIR guided implementation design [26]. We used the “Learning Collaborative” [37] method to help stakeholders design center-specific approaches to surmounting barriers to HKTP implementation to achieve healthcare quality improvement [38]. Learning collaborative discussions occurred via one teleconference call in September 2016 and one 2-day in-person meeting at Northwestern University in October 2016 (Fig. 1). Four teleconference calls occurred in 2017, in which stakeholders discussed successes and challenges to implementation strategies, modifications made, and strategized solutions for implementing modifications at their institutions. Additionally, weekly and biweekly telephone calls were conducted with each site’s Research Coordinators, and on occasion other stakeholders (e.g., Site PIs, administrators, outreach staff), to ascertain intervention progress and modifications.

### Data collection

Site visits were conducted in May 2016 and March–April 2017. In-depth, in-person, one-on-one and group interviews were conducted with transplant stakeholders by a trained female social scientist with a PhD, co-PI (EJG), using the CFIR Interview Guide [39]. An additional file presents the interview questions (see Additional file 2). Interviews assessed stakeholders’ attitudes about the implementation, perceived provider, operational, and center-level barriers and facilitators to HKTP implementation, and the intervention’s modifications. Additionally, group discussions, led jointly by EJG and surgeon co-PI (JCC), assessed operational logistics to intervention implementation and were not guided by CFIR. Interviews and discussions were audio-recorded and lasted 30–60 min. Learning collaborative discussions were audio-recorded. While the first learning collaborative telephone meeting lasted 2 h, the second learning collaborative meeting was a 2-day in-person workshop, and the remaining telephone meetings lasted 1 h. Handwritten field notes, including reflexive reflections, were taken by one co-PI (EJG) and/or Research Coordinators during research activities. Thus, modifications, as described by both sites, were prospectively documented.

**Fig. 2:**
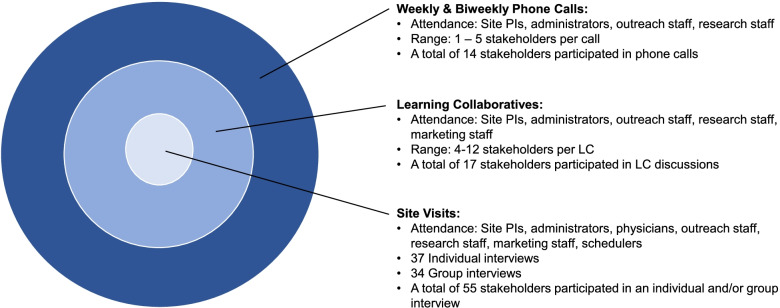
Data collection approaches by frequency

Figure 2 illustrates 3 concentric circles, with each circle representing a different data collection activity, the frequency of which is denoted by the circle size. The weekly/biweekly meetings occurred most frequently, followed by learning collaborative meetings and site visits. The role and number of stakeholders overlapped across data collection activities. For example, site PIs, administrators, outreach staff, and research staff attended weekly/biweekly calls, learning collaborative meetings, and participated in site visits interviews.

### Qualitative analysis

#### 2016 CFIR analysis

Transcriptions of 2016 data collected underwent a thematic analysis of barriers and facilitators to carrying out the HKTP during the pre-implementation phase, as described elsewhere [31]. Briefly, we used CFIR constructs as codes that guided the thematic analysis using the constant comparative [40], deductive, and inductive coding methods [41, 42]. In this paper, we only report the results of the deductive coding. After achieving inter-rater reliability (kappa > 0.80) [43], multiple teams of two coders discussed discrepancies and achieved consensus. The CFIR interview guide was used to conduct in-person, one-on-one, and group interviews during 2016 and 2017. However, we did not perform CFIR thematic analysis of the interview data collected in the first year of implementation (2017). Thus, the 2016 data were used to generate the comparative site descriptions.

#### 2017 FRAME analysis

We performed a framework analysis using the FRAME by coding transcriptions of the audio-recordings and field notes from the implementation phase (2017) for the presence of modifications, their nature, initiator, intervention component, and intent. A codebook was based on Stirman’s FRAME. The research team (EJG, MS, ER, NA, JU) held multiple 1-h-long analytic retreats to review independently coded transcripts and revise the codebook for clarity, in an iterative process. The four-member coding team reached inter-rater reliability (kappa > 0.60), then resolved discrepancies between coders through discussion [31, 41, 42]. All transcripts were uploaded into qualitative analysis software (MAXQDA v.12).

#### Axial coding

During the second phase of analysis, axial coding techniques from grounded theory [44] were used to examine the relationship between CFIR factors and modifications. This process allowed us to theorize how CFIR contextual factors during the pre-implementation phase may have generated causal conditions prompting the implementation phase modifications. The research team had previously used thematic analysis [45, 46] to code all expressions of CFIR framework domains in transcripts of interviews conducted during the pre-implementation period [31]. Memo summaries of themes about each CFIR domain were examined for relationships with the modifications. We excluded the types of modifications that manifested ≤ 5 occurrences (i.e., repeating elements, shortening) from comparative analysis because of the insufficient number of examples. Both site PIs were provided the transcript of results for review.

## Results

### Demographics

Fifty-seven stakeholders (Site-A: *n* = 26, Site-B: *n* = 31; 100% recruitment rate) participated in one or more research activities, including 37 in-depth, one-on-one interviews (*n* = 28, some participated in interviews twice, once in 2016 and again in 2017), 34 group discussions (*n* = 45), and/or 6 learning collaborative discussions (*n* = 17) (Table 1). Most participants were female (61%), non-Hispanic (70%), and included non-physician clinicians (nurses, social workers) (25%), physicians (19%), administrators (14%), and staff (e.g., schedulers, marketing, IT, and research) (42%).

**Table 1:** Participants’ demographic characteristics by study site

Characteristic	Total, *N* (%) *N* = 57	Site-A, *N* (%) *N* = 26^a^	Site-B, *N* (%) *N* = 31^b^
Gender			
Female	35 (61)	15 (58)	20 (65)
Male	22 (39)	11 (42)	11 (35)
Ethnicity			
Non-Hispanic	40 (70)	16 (62)	24 (77)
Hispanic	17 (30)	10 (38)	7 (23)
Training			
Non-physician clinician	14 (25)	4 (15)	10 (32)
Physician	11 (19)	6 (23)	5 (16)
Administrator	8 (14)	4 (15)	4 (13)
Staff: Marketing	7 (12)	2 (8)	5 (16)
Staff: Scheduler	5 (9)	2 (8)	3 (10)
Staff: Other^c^	12 (21)	8 (31)	4 (13)

^a^Site-A had 22 participants in 2016, and 13 in 2017. The total represents the number of unique participants

^b^Site-B had 29 participants in 2016, and 16 in 2017. The total represents the number of unique participants

^c^Other = Front desk, research, and IT staff

### Comparative site descriptions

The following descriptions of the intervention sites are essential for obtaining valid measures of the effects of modifications on desired outcomes [23]. Table 2 compares both sites in light of CFIR constructs.

**Table 2:** Study site descriptions by consolidated framework for implementation research domains and constructs^a^

DOMAIN, Construct		Brief description	Study site	
			A	B
**I. Intervention characteristics**				
A	Evidence Strength & Quality	“Stakeholders’ perceptions of the quality and validity of evidence supporting the belief that the intervention will have desired outcomes.” (p. 6)	Generally lacks but one administrator has knowledge	Lacks knowledge
B	Relative Advantage	“Stakeholders’ perception of the advantage of implementing the intervention versus an alternative solution.” (p. 6)	Less perceived relative advantage because of similar programs	Greater perceived relative advantage because of lower living kidney donation numbers
C	Complexity	“Perceived difficulty of implementation, reflected by duration, scope, radicalness, disruptiveness, centrality, and intricacy and number of steps required to implement.” (p. 6)	This is a complex intervention in terms of involving multiple interacting intervention components, stakeholders, and interactions with different groups of patients (see Additional file 1)	
**II. Outer setting**				
A	Patient Needs & Resources	“The extent to which patient needs, as well as barriers and facilitators to meet those needs, are accurately known and prioritized by the organization.” (p. 7)	Similar perception	
B	Cosmopolitanism	“The degree to which an organization is networked with other external organizations.” (p. 7)	Similar level	
**III. Inner setting**				
A	Structural Characteristics	“The social architecture, age, maturity, and size of an organization.” (p. 7)	Similarly large and high volume	
B	Networks & Communications^b^	“The nature and quality of webs of social networks and the nature and quality of formal and informal communications within an organization.” (p. 8)	The Site-A collaboration with the community nephrology organization was a challenge, but posed little red tape	Bureaucratic red tape and relationships with the "mothership"
C	Culture	“Norms, values, and basic assumptions of a given organization.” (p. 8)	Decision making is centralized at the transplant division level	Decision making is diffused throughout the enterprise, which values physician led initiatives
D	Implementation Climate	“The absorptive capacity for change, shared receptivity of involved individuals to an intervention, and the extent to which use of that intervention will be rewarded, supported, and expected within their organization.” (p.8)		
1	Tension for Change	“The degree to which stakeholders perceive the current situation as intolerable or needing change.” (p. 8)	Less need for change because other similar programs are perceived as good	Greater perceived need for change because of lower living kidney donation numbers
2	Compatibility	"The degree of tangible fit between meaning and values attached to the intervention by involved individuals, how those align with individuals’ own norms, values, and perceived risks and needs, and how the intervention fits with existing workflows and systems.” (p. 8)	Greater compatibility with HKTP because of similar programs in place, and because a greater number of resources were available to make this work. Similar marketing challenge	Greater compatibility challenge because no similar program existed and a large change to current workflow. Similar marketing challenge
3	Relative Priority	“Individuals’ shared perception of the importance of the implementation within the organization.” (p. 8)	Some competing priorities, particularly in the implementation of human resources system update	Greater competing priorities, including Breeze updates that require coordination of all three enterprise sites
4	Goals and Feedback	“The degree to which goals are clearly communicated, acted upon, and fed back to staff, and alignment of that feedback with goals.” (p. 9)	Comparable levels	
5	Learning Climate	“A climate in which: a) leaders express their own fallibility and need for team members’ assistance and input; b) team members feel that they are essential, valued, and knowledgeable partners in the change process; c) individuals feel psychologically safe to try new methods; and d) there is sufficient time and space for reflective thinking and evaluation.” (p. 9)	Differing levels of seniority on large volume of providers	More team-based culture
E	Readiness for Implementation^c^	“Tangible and immediate indicators of organizational commitment to its decision to implement an intervention.” (p. 9)	Similar support commitment	
1	Leadership Engagement	"Commitment, involvement, and accountability of leaders and managers with the implementation.” (p. 9)	Greater leadership support	More diffused leadership support.
2	Available Resources	“The level of resources dedicated for implementation and ongoing operations, including money, training, education, physical space, and time.” (p. 9)	Greater resources including: space, time, education materials in Spanish, Spanish-speaking staff (initially lacked coordinator and marketing budget)	Fewer resources including: overwork of staff, lack of Spanish-speaking staff, lack of capacity to handle greater patient volume, website and patient materials not in Spanish, space difficult to schedule, and general perception of lack of time
**IV. Characteristics of individuals**				
A	Knowledge & Beliefs about the Intervention^d^	“Individuals’ attitudes toward and value placed on the intervention as well as familiarity with facts, truths, and principles related to the intervention.” (p. 9)	Hispanic identity, academic goals, and perceptions of underserved group motivated stakeholders to deploy the intervention	
B	Self-efficacy^e^	“Individual belief in their own capabilities to execute courses of action to achieve implementation goals.” (p. 9)	Greater self-efficacy because more experience implementing similar organizational-level interventions. They had more confidence in being able to do it	Less experience implementing similarly sized interventions
C	Individual Stage of Change^f^	“Characterization of the phase an individual is in, as he or she progresses toward skilled, enthusiastic, and sustained use of the intervention.” (p. 10)	Similar levels of change commitment	

^a^As Damschroder and colleagues [25] note, implementation science researchers must discern which of the CFIR constructs are most appropriate for their context and study aims. Because the aims of this paper were to compare two sites, using the same intervention, we excluded several factors related to the nature of the intervention (e.g., intervention source, adaptability, trialability, design quality and packaging, and cost). In addition, because the intervention occurred at the organizational level, we did not consider few outer setting characteristics (e.g., external policy and incentives). CFIR constructs that sites exhibited in a comparable manner were noted above

^b^We analyzed “Networks and Communications” as a feature of Structure in our coding scheme

^c^We referred to “Readiness for Implementation” as support in our coding scheme

^d^We referred to “Knowledge & Beliefs about the Intervention” as motivation in our coding scheme

^e^We referred to “Self-efficacy” as an organization’s prior experience with interventions in our coding scheme because individuals do not typically implement organizational-level interventions

^f^We referred to “Individual Stage of Change” as change commitment in our coding scheme

#### Site-A

Site-A was part of an enterprise system whereby surgeons practiced at one of two of the institution’s locations within the state, with Site-A serving as the larger, dominant location. While Site-A performed kidney transplant surgeries and provided post-transplant care, all pre-kidney evaluations were outsourced to a community nephrology organization, with which Site-A has had a long-standing collaborative relationship. Accordingly, intervention implementation required collaboration between staff, administrators, and surgeons from Site-A and the community nephrology organization.

Site-A is comprised of experienced surgeons following a team-based approach, whereby collaboration from all team members was expected. Decision making was centralized at the transplant division level, which enabled quick changes and approvals. The HKTP was led by the Site-A lead administrators and the site PI. Because the community nephrology organization was outside of Site-A’s charge, challenges arose in ensuring the HKTP’s successful implementation. While the HKTP generally fit well within Site-A, and there was ample space to deliver the education sessions, hiring a bicultural/bilingual outreach staff was a protracted process.

#### Site-B

Site-B was also part of an enterprise system involving three integrated campuses across the country, but Site-B was not the leading location. All institutional changes required formal review and approval by the leading institutional location before changes could be implemented across campuses. Further, Site-B had different *structural characteristics*—marked by greater complexity due to the collaboration between two different clinical departments to enable a Hispanic physician to deliver the HKTP education sessions in Spanish.

Site-B can be characterized as having an egalitarian culture, whereby physicians and administrators were expected to co-lead institutional initiatives. Decisions are commonly protracted because they are made through multiple committee meetings involving physicians and staff. While the HKTP generally fit well within Site-B, finding the space to deliver the education sessions remained an ongoing challenge. Although there was a bilingual outreach staff to deliver the dialysis lobby days, this staff member was constrained by other institutional responsibilities that limited time for delivering the intervention.

### Modifications

Site-B made nearly twice as many modifications than Site-A (*n* = 29 versus *n* = 18) (Table 3). Sites differed in the proportions of *delaying/skipping* (Site-A 50% versus Site-B 28%) and *adding* (Site-A 11% versus Site-B 28%), but were comparable in *substituting* (Site-A 17% versus Site-B 17%) and *tweaking* (Site-A 17% versus Site-B 14%) modification types.

**Table 3:** Frequency of modification initiator, type, and goal by study site

	Total ***N*** (%)	Site-A ***n*** (%)	Site-B ***n*** (%)
**Number of overall modifications**	47 (100)	18 (38.3)	29 (61.7)
**Modification initiator**			
Transplant team	30 (64)	12 (66)	18 (62)
Individual	9 (19)	3 (17)	6 (21)
Institution	6 (13)	3 (17)	3 (10)
More than one	2 (4)	0	2 (7)^a^
**Modification type**			
Removing/skipping/delaying	17 (36)	9 (50)	8 (28)
Adding	10 (21)	2 (11)	8 (28)
Substituting	8 (17)	3 (17)	5 (17)
Tailoring/tweaking/refining	7 (15)	3 (17)	4 (14)
Spreading	2 (4)	1 (6)	1 (3)
Shortening/condensing	1 (2)	0	1 (3)
Repeating elements	1 (2)	0	1 (3)
Reordering	1 (2)	0	1 (3)
Integrating another treatment into the intervention	0	0	0
Lengthening/extending	0	0	0
Loosening structure	0	0	0
Changes in packaging or materials	0	0	0
Drift	0	0	0
**Modification goal**			
Improve effectiveness/outcomes	5 (11)	2 (11)	3 (10)
To address cultural factors	4 (9)	2 (11)	2 (7)
Increase reach or engagement	4 (9)	1 (6)	3 (10)
Improve feasibility	4 (9)	1 (6)	3 (10)
Improve fit with recipients	2 (4)	1 (6)	1 (3)
Increase satisfaction	0	0	0
Increase retention	0	0	0
Reduce cost	0	0	0
Not applicable^b^	28 (60)	11 (61)	17 (59)

^a^Two modifications were initiated by both the institution and an individual

^b^Twenty-eight modifications did not have a clear goal or did not fit under recommended FRAME goals

Across sites, the *transplant team* consistently initiated most modifications (Site-A 66%; Site-B 62%). *Individuals* instigated proportionately slightly more modifications at Site-B (21% versus Site-A 17%). In contrast, *institutions* instigated proportionately slightly more modifications at Site-A (17% versus Site-B 10%). At Site-B, both the *institution* and *individuals* initiated two modifications (7%).

The goals of making modifications slightly varied by site (Table 3). Sites were comparable in making modifications that aimed to improve effectiveness/outcomes (Site-A 11%; Site-B 10%). However, the goal of addressing cultural factors drove proportionately more modifications at Site-A (11% versus Site-B 7%), whereas the goals of increasing reach and improving feasibility drove proportionately more modifications at Site-B (10% versus Site-A 6%). The goals of increasing satisfaction and retention or reducing costs did not motivate any modifications across sites.

### Modification similarities between sites

#### Modification types

**Skipping and delaying**

Both sites made similar *skipping*-type modifications, primarily due to their institutions’ *structural characteristics.* For example, both sites did not establish a direct, Spanish-speaking telephone line in the transplant division, per the protocol. Because Site-A worked in collaboration with a community nephrology organization that made and received all HKTP-related intake calls in which “everybody who answers the phone is bilingual,” Site-A did not perceive any problems in providing access to the HKTP to their Spanish-speaking patients. Site-B did not implement a Spanish telephone line because doing so would require a large infrastructural change that would challenge the institution’s “philosophy… [to] provide the same care at every site no matter what door you are walking into.” As a stakeholder from Site-B explained, setting up the Spanish line would be a “big ask for the institution” because “the Spanish portion of them having access to someone who speaks Spanish has never been an option at the institution”. Thus, if a Spanish-speaking telephone line were implemented in the transplant division, the institution would have to implement one in other clinical divisions.

Both sites experienced delays in publishing the Spanish HKTP information on their institutional websites due to *structural characteristics*. Site-A encountered delays in translating the HKTP website content because its merger with another healthcare institution posed other institutional priorities. Site-B encountered delays because it had not received approval from institutional enterprise leadership. As one interviewee explained, “Well, I know that our website needs to be translated and unfortunately I have no control over that. This is something that needs to be done by [the enterprise]”.

Both institutions delayed the translation of HKTP patient-facing materials (i.e., HKTP brochures, patient letters, flyers) into Spanish. Both institutions attributed these delays, in part, to their lack of Spanish-speaking staff or time available by such staff to dedicate to this task (*available resources*). As one interviewee from Site-B explained, “I mean we have international services that does translation for our patients, and I’ve asked them before if they would translate information. I think in the past when they are more heavily staffed they were able to, but now they’re short staffed, and they’re not able to.” Delays were also due to conflicts in coordinating with the transplant team, translation services, and marketing department. The intervention’s 16 components, involving different institutional departments and different organizations, made skipping and delays more likely to arise, given the challenges of coordination between these organizational units.

Both sites also skipped monitoring their progress and/or success with the intervention, which coincides with the CFIR construct of *implementation climate* in terms of *goals and feedback* systems. Thus, sites were equally unaware of their HKTP participants’ status in terms of numbers, progress through the LDKT evaluation process, and outcomes. Stakeholders at both sites acknowledged early on that they did not evaluate their program by patient ethnicity or race. One interviewee from Site-A explained, “I wish I had a number, I should, I know how much you guys have a better idea of your numbers, and our numbers than we did, but I think… just for me, I look around the community, and I’m like this is obviously a huge need.” An interviewee at Site-B explained further “I don’t think ever looked at 'Oh let’s look at our Hispanic population of recipients!' How many living donors do they have? Did anyone call in? At what point? How far do they make through the process? Did they withdraw? Were they excluded? Like I think that historically, we never really looked at that.”

**Adding**

Both sites added an intervention component of providing laptops to potential living donors during the HKTP clinic to facilitate Spanish-speaking potential living donors’ access to and completion of the English-language living donor online medical history questionnaire, which was not available in Spanish.

**Substituting**

Both sites made similar substituting type of modifications to the intervention, which appeared to compensate for the lack of *available resources.* To increase the HKTP reach to the Hispanic patient population, the bilingual/bicultural research coordinators at both sites occasionally assisted the non-Hispanic white outreach staff when visiting dialysis centers with a large Hispanic population. Further, at Site-A, there was originally no bilingual/bicultural outreach staff; therefore, they relied on an English-speaking outreach staff. For example, at Site-A, one stakeholder stated “…we’ve had a position posted for essentially 9 months, and while we have had many, many applicants, none, we’ve interviewed several people, and it has not been a good match. … And so [Ms. X], who is here with us today too, is our outreach coordinator, and she’s absolutely promoting this, and [the bicultural/bilingual research coordinator] is helping some. But we still don’t have this full-time dedicated person.” At Site-B, a bilingual/bicultural transplant nurse delivered the HKTP education sessions when bilingual/bicultural transplant physicians were unavailable.

**Tailoring, Tweaking, Refining**

Both sites performed tweaking-type modifications to the HKTP to better meet the needs of their patient population. For example, both transplant teams modified the schedulers’ script because the schedulers had expressed that patients perceived the script’s delivery as forced and rehearsed, rather than natural and organic.

**Spreading**

At both sites, scheduling conflicts arose due to limited staffing availability and patients’ needs. At Site-A, the absence of enough dietitians on HKTP clinic days made scheduling patients with HKTP physicians a challenge. At Site-B, multiple HKTP patients were seen by nephrologists who simultaneously needed an interpreter, but there were insufficient numbers of interpreters and the nephrologists did not like using the interpreter app on iPads. These staffing conflicts resulted in patients' appointments with nephrologists to be spread out, which led to some HKTP patient evaluation clinics being extended over 2 days, instead of occurring in 1 day, as recommended in the intervention protocol.

### Modification differences between sites

CFIR Inner Setting factors in the implementation preparation phase explained many differences in the number and types of modifications made across sites during the implementation phase. Details including illustrative quotations about each modification made by site are presented in the Supplemental File 3. Site-A’s prior experience and success with implementing other interventions increased the institution’s confidence in implementing the HKTP (i.e., their collective *self-efficacy*). Site-A had previously expanded their services to living donors, modeled after a program they adopted from another institution. “[This other institution has] a red phone and when donors call, it’s always answered. [So we implemented], a phone number that is always answered also, but the focus was, we value you as a donor, and we are going to take excellent care of you … I mean that project, we implemented and brought it in 2½ years ago.” Site-A’s previously successful implementation experience led them to make fewer overall modifications. The greater number of intervention modifications at Site-B was due to the HKTP being less *compatible* with existing workflows, *structural characteristics*, *culture*, and *available resources*. One interviewee explained, “I think that what it’s gonna make it hard to implement is that nothing happens at [Site-B] without 20 meetings and 20 people dotting the I and crossing the T. I think the biggest barrier is just kind-of going through the process, and then redoing the process, and making sure that everyone is part of the process and I think that it’s kind of like the nail in the hay.” For example, Site-B’s HKTP outreach staff had multiple work commitments, which prevented them from performing the minimum 16 h of outreach per week for a total of 832 h for the year, as outlined in the intervention protocol. Further, Site-B could not schedule the target number of patients to attend the HKTP education sessions because of limits imposed by the size of the education room.

#### Modification types

**Skipping and delaying**

Site-A delayed or skipped proportionately more intervention components than Site-B, which can be explained by the CFIR inner factor of *structural characteristics*. Site-A had to coordinate the implementation of HKTP components with their partnering community nephrologist organization, which had their own rules and leadership. As one interviewee explained, “I think that one thing that makes it a little more difficult is that our recipient [and] donor teams are in two different locations with two different staff, so it’s almost like we have to have double staff if, you will. So I think in general that makes it a little more difficult, because of the contractual agreement that we have with [second organization] for the recipient portion, while we collaborate and we pay them contractually, we don’t manage them, and so [they] have recipients, I have donors.” Thus, despite their good working relationship, Site-A was not able to ensure that the external community nephrology organization could translate patient letters into Spanish or mail letters to nephrologists on time. Moreover, Site-A’s merger with another healthcare organization delayed the translation of HKTP information to be posted on the institutional website.

By contrast, Site-B’s egalitarian *culture* resulted in diffused *leadership engagement* in the intervention. One interviewee noted concerns that Site-B’s PI did not speak Spanish and that another doctor from the Urology department would be delivering the Spanish education sessions. Site-B’s PI did not exert their upward influence (also known as the “Pelz effect” [47]) on institutional committees to expedite their review and approval of essential HKTP components. Stakeholders at Site-B attributed delayed approvals of intervention components, including the HKTP Spanish website content and educational materials’ approval, to their institution’s time-consuming decision-making process.

**Adding**

Site-B added proportionately more intervention components than Site-A. Most of Site-B’s modifications involved adding elements to the intervention, which were related to the institution’s *culture*. One interviewee explained, “You don’t really have power. You don’t really go around telling people what to do. You just find ways to identify key people and stakeholders and help motivate them to do the job. We don’t really run an institution of authoritative dictatorship. It’s not like a traditional academic environment where the Chair says, 'Okay, this is what you all are going to do' and people say, 'Okay [the Chair] told me to do that.' It doesn’t work that way here.” The team-based approach to decision making placed a high value on obtaining input from all levels within Site-B’s social structure. Stakeholders involved in HKTP implementation behaved as though they felt empowered to make additions that they believed would improve the HKTP that bypassed the prolonged team-based approach to decision making. Additions included the bilingual outreach staff creating a flyer that was posted in dialysis centers to advertise the upcoming lobby days, and the bilingual/bicultural research staff creating a folder containing educational materials to give to potential living donors and family members attending the HKTP clinic. By contrast, decision making at Site-A was concentrated at the transplant division level, which facilitated approvals with implementing the HKTP with greater efficiency.

Site-A’s *culture* featuring strong *leadership engagement* by the transplant division facilitated approvals with implementing the HKTP with greater efficiency, leading to proportionately fewer components of the intervention being added. One interviewee explained, “We are organized in a fashion, so for us, we do whatever we want. There is no one who will oppose us. I mean no one will say no. If we say this is what we want to do, then everybody else says, 'Yes, sir.' That’s the way it works.” The strong *leadership engagement* from operation administrators at Site-A fostered momentum in intervention implementation.

#### Modification initiator

The modifications made by individuals at Site-B were primarily related to the institutional *structural characteristics*, *culture*, and absence of sufficient *leadership engagement*. As described above, decision making typically required multiple team meetings to come to a consensus. For example, the HKTP flyer developed for distribution to dialysis patients during outreach visits had to undergo an extensive chain of review before receiving authorization from the institutional enterprise’s “mothership.” Site-B’s team-based *culture* and the need to rapidly implement the intervention enabled individual stakeholders to expedite modifications by bypassing the typical lengthy approval process through “workarounds.” By contrast, decisions at Site-A were made internally by the transplant team. For example, the transplant team decided to host their usual Spanish Town Hall Question and Answer discussions for the public to promote the HKTP, adjusted the day and time of HKTP clinics to solve scheduling conflicts with healthcare providers, and cancelled HKTP clinics when too few patients were scheduled.

## Discussion

In this study of a complex, culturally competent care intervention, we found that both institutions made numerous modifications. Modifications occurred after the intervention had been implemented and as new organizational issues emerged. By contrast, Stirman’s review found that most studies described modifications made proactively, early in the implementation process [4]. Thus, our findings underscore the importance of assessing modifications throughout the implementation phase.

This study is among the first to identify the CFIR factors that influence implementation outcomes, a new area of CFIR research [16]. CFIR Inner Setting factors identified during the pre-implementation period helped explain the types of modifications made. The CFIR factor, *structural characteristics*, appeared to contribute to intervention delays/skipping in Site-A. By contrast, the lack of *leadership engagement* appeared to contribute to delays/skipping in Site-B. These examples illustrate how institutional-level factors are relevant to modifications made to complex interventions.

Modifying interventions is the subject of much debate because they potentially interfere with implementation fidelity [14]. Our findings suggest that CFIR factors influenced the type and number of modifications that institutions made to the HKTP intervention, which is an implementation outcome. Based on our previous research [27], a greater number of modifications was associated with reduced intervention effectiveness. Thus, this research connects CFIR factors, implementation, and intervention effectiveness. Our findings may help explain what different organizational factors lead to modifications in intervention implementation, which may help institutions to adapt the intervention before implementation and to more effectively foster its implementation [48].

Most modifications involved delaying/skipping, adding, or tailoring/tweaking intervention components, consistent with other research [23]. Our findings shed light on CFIR factors that explain modification types that may arise during implementation. Specifically, we found that Inner Setting factors such as *culture*, *available resources*, *compatibility,* and *goals and feedback* largely explain these modifications. The sites’ nested layers of institutional bureaucracy (i.e., *structural characteristics*) promoted skipping and delaying modification types.

Most modifications were made by the transplant team (64%), suggesting a unified or thoughtful approach. However, 19% of modifications were made by individuals, which highlights the need for greater oversight of the intervention implementation process by study champions. Modifications that originated from individuals were most common when there was an egalitarian culture, suggesting that such intervention sites require greater oversight than sites with greater *leadership engagement*.

### Practical implications

Our findings have practical implications for leaders intending to implement complex interventions. In particular, our study sheds light on Pawson and Tilley’s pivotal question, “What works, for whom, in what circumstances, in what respects and why?” [49] We found that when there is greater compatibility between an intervention and the institution, and when the institution possesses ample resources to support the intervention, fewer modifications were made. Similarly, when the institution maintains an egalitarian form of culture, more additions were made to the intervention. Moreover, when an institution has complex organizational structures (*structural characteristics*), greater delays to implementation occurred. Practically, these findings can help prepare interventionists to expect certain modifications in certain settings.

### Strengths

Our study’s strengths include a prospective analysis of a complex intervention. We evaluated the implementation at the organizational level at two geographically distinct healthcare organizations. Given that the HKTP was devised as a culturally competent intervention, our evaluation of modifications to the HKTP largely controls for concerns raised by the cultural adaptation field within implementation science, modifications based on clients’ cultural backgrounds, and enables a broad analysis of organizational factors influencing modification. Involving the co-PI (EJG), who had an intimate knowledge of the intervention and what comprised minor versus moderate modifications, helped ensure appropriate modification classification in the coding and analysis processes.

### Limitations

Our study has limitations. Detailed descriptions of modifications depended on self-report, which may have been limited by social desirability bias. However, triangulation through interviews with multiple stakeholders and member checking through weekly phone calls with Site-A and Site-B research staff and quarterly learning collaborative calls served to validate and clarify the nature of modifications. This analysis did not evaluate the impact of modifications on implementation fidelity and on HKTP outcomes. The relative weight of modifications for their impact on fidelity to different intervention components remains to be determined [17].

We subsequently evaluated the intervention’s sustainability in light of modifications made over time [50, 51]. Sustainability, as opposed to effectiveness, describes the degree to which the intervention is continued over time. Sustainability is particularly challenging for complex interventions, like the HKTP [52].

## Conclusions

Complex interventions require the coordination of multiple departments and individuals. The implementation of complex interventions creates many opportunities for modifications to occur in various locations within the organization. This research demonstrates that identifiable factors from the CFIR explain the frequency, type, and source of those modifications. In doing so, it guides how organizational actors are likely to modify the intervention, either to better fit the unique context or in ways that undermine intervention fidelity.

## Supplementary information

**Additional file 1.** HKTP Implementation Components (same as protocol table). This file describes all 16 HKTP intervention components.

**Additional file 2.** 2017 Site Visit Interview Guide for the Site PI and Administrators. This file provides the interview guide used for the 2017 site visits.

**Additional file 3.** Modification Description, Reason, Illustrative Quotation, Modification Type, and Initiator by Study Site. This file presents a description of modifications, the reasons why they occurred, modification type, and illustrative quotations by study site.

## Data Availability

The datasets generated and/or analyzed during the current study are not publicly available due to the sensitive nature in that individual privacy could be compromised.

## Abbreviations

CFIR: Consolidated Framework for Implementation Research
Co-PI: Co-Principal Investigator
FRAME: Framework for Reporting Adaptations and Modifications-Expanded
HKTP: Hispanic Kidney Transplant Program
LDKT: Living Donor Kidney Transplantation
NHW: Non-Hispanic White
NM: Northwestern Medicine®
