# Supplementary material for: Implementation and modification of an organizational-level intervention: a prospective analysis
Source: Implement Sci Commun. 2022 Jun 3;3:59. doi: 10.1186/s43058-022-00296-0 (PMC9164326; doi:10.1186/s43058-022-00296-0)
Supplement: Supplementary file 1 — Additional file 1. HKTP Implementation Components (same as protocol table). This file describes all 16 HKTP intervention components. [file 43058_2022_296_MOESM1_ESM.docx]

| **Additional file 1. HKTP Implementation Components*** | |
| --- | --- |
| **Intervention Component ID #** | **Intervention Component Description** |
| *For patients at the dialysis center:* | |
| 1 | At the dialysis center, the bilingual (bicultural) outreach staff hold lobby days to introduce, hand out an information folder in Spanish about the HKTP, and invite Hispanic patients to attend the HKTP. Staff provide patient contact information to clinic schedulers to set up a clinic visit for each patient. |
| 2 | Transplant center telephone bilingual* schedulers offer (follow the script) the HKTP clinic as an option to all Hispanic patients calling to schedule transplant evaluation, regardless of the patient’s spoken language. Schedulers encourage patients to bring their family members to the HKTP visit. |
| 3 | Establish a Spanish speaking telephone line at the transplant center |
| 4 | The transplant center’s phone line offers (live) Spanish-speaking schedulers when patients call. |
| *For patients and family who visit the HKTP:* | |
| 5 | Bicultural Transplant surgeon delivers the 2 back-to-back HKTP education sessions that provide same care as the English program as well as address cultural concerns about transplantation in Spanish |
| 6 | Education sessions address Hispanic cultural and religious concerns & myths |
| 7 | Education sessions include family members in both sessions |
| 8 | Bicultural transplant surgeon or bilingual transplant nephrologist holds a ‘wrap up’ clinical visit with each patient and asks about available potential living donors. |
| 9 | HKTP bilingual clinical staff and/or outreach staff contact patients who have not completed evaluation within 10-12 weeks |
| 10 | HKTP Director calls patients and referring MD if patients have not completed pre-transplant evaluation by 8-10 and 12 weeks |
| 11 | Availability of interpreters or interpreter services |
| 12 | Data collection on patients in a transplant database for quality assurance and identifying areas for improvement |
| *For the local nephrology community:* | |
| 13 | Letters are mailed to nephrologists introducing the HKTP to increase patient referral |
| 14 | Transplant center holds in-service presentations to local social worker and nephrology organizations to increase awareness of the HKTP to increase patient referral. |
| *For the public:* | |
| 15 | Transplant center engages in marketing events (e.g., interviews in news and social media about the HKTP directed to the Hispanic community in Spanish (TV, radio, newspaper). |
| 16 | Transplant center website hosts information about the HKTP in Spanish |
| *Source: Adapted from Gordon et al. Protocol. BMC Health Services Research 2018. | |
