# Supplementary material for: Implementation and modification of an organizational-level intervention: a prospective analysis
Source: Implement Sci Commun. 2022 Jun 3;3:59. doi: 10.1186/s43058-022-00296-0 (PMC9164326; doi:10.1186/s43058-022-00296-0)
Supplement: Supplementary file 2 — Additional file 2. 2017 Site Visit Interview Guide for the Site PI and Administrators. This file provides the interview guide used for the 2017 site visits. [file 43058_2022_296_MOESM2_ESM.docx]

**Additional file 2. 2017 Site Visit Interview Guide for the Site PI and Administrators**

**HKTP Clinic Overall (Site PIs, Administrators/Operations)**

Role-Specific Updates

Overall, how is the HKTP program going?

How is the HKTP clinic running?

What kind of interactions do you have with patients in the HKTP clinic (outside the education session)?

What seems to be working well with the HKTP program/clinic? How can you tell?

What seems to not be working well with the HKTP program/clinic?

I know that you’ve committed a lot of time into this program. Since the HKTP program has been in place, what has your time investment been like (more/less) (since December 2016/January 2017)?

What marketing strategies have worked?

- What is the process like for sending out the referral letters to nephrologists?

How is the BIA data collection process going?

CFIR/Intervention Characteristics: Adaptability

What are some of the challenges to running the HKTP clinic and program overall?

- How have you dealt with these challenges to make the HKTP fit better in your institution?
- How are these changes working out for you?

What kinds of changes do you think you will (still) need to make to the implementation so it will work more effectively in your setting? Will those changes be do-able?

CFIR/Inner Setting/Structural Characteristics

What kinds of infrastructure changes are still needed to accommodate the intervention?

- How will you work around those structural challenges?

EVIDENCE: Patient/family/donor Experiences (also CFIR Outer Setting)

What kind of positive feedback about the HKTP have you heard?

What kind of negative feedback about the HKTP have you heard?

- Have you elicited information from patients/families about their experiences with the HKTP? Are you documenting or tracking patients’/families’ feedback?
- What are their perceptions of the intervention? What have you heard?
- To what degree is the HKTP congruent with patients’/families’ preferences for culturally competent care?

EVIDENCE: Knowledge/Clinical Experience

Based on your clinical experience with the HKTP clinics so far, what evidence do you see that the HKTP is working?

Based on your perceptions of the HKTP clinics, what evidence do you see that the HKTP is working? (administrators only)

EVIDENCE: Relative Advantage

Compared to before the HKTP program, what advantage do you see the HKTP program offering your institution?

- To what extent do you believe that the HKTP program is improving clinical practice/care for Hispanics (key stakeholders)

EVIDENCE: Compatibility

How well does the HKTP fit with your organizational priorities and values and norms?

- How well does the HKTP fit with existing work processes and practices in your setting?
- To what extent do you believe the HKTP can increase the number of Hispanic potential recipients/ donors getting evaluated, and the number of potential living donors?

EVIDENCE: Complexity

How hard is it going to be to sustain the HKTP program?

To what extent can the HKTP be easily described to others outside of your unit/department, and across the transplant center and hospital?

How committed is the study site team to maintaining the HKTP?

CFIR/Inner Setting: Relative Priority

To what extent might the HKTP take a backseat to other high-priority initiatives going on now? (administrators only)

How are you/the educators juggling competing priorities in your own work? (PI only)

How will people involved in implementing the protocol be held accountable to sticking to the action plan?

CONTEXT: Receptivity

**What other priorities or initiatives might interfere with the HKTP because they will take a great deal of energy or because they are happening at the same time as the HKTP**

To what extent does the physical location, room size or configuration meet the needs of the intervention?

To what extent do communication channels or formal networks exist for staff to obtain feedback and provide input regarding the HKTP program?

To what extent do you/key stakeholders have the power and authority to carry out expected tasks of change required by the HKTP program?

CONTEXT: Culture

How might the HKTP be challenging your organization’s culture? Staff culture?

How has your practice previously sought to address cultural differences among patients?

In what ways is HKTP consistent with or different from that?

How well is your team communicating and collaborating amongst all those involved across units/disciplines to implement the HKTP? Can you give examples of what is/is not working?

CONTEXT: Evaluation (capacities/systems already in place for collecting data on the intervention)

What systems still have to be set up?

- Has Breeze been fully implemented in Spanish? How is that working out?
- How is the system for identifying potential donors who were medically ruled out working out?
- What kind of data is your organization collecting on the HKTP in how well its doing or working?

FACILITATION

To what extent is the facilitator providing help and support to achieve specific goals (e.g., problem solving to resolve a specific issue)?

- To what extent is the facilitator helping teams to analyze and reflect and change their own attitudes, behaviors and ways of working, related to the HKTP intervention?

CONTEXT: Leadership

To what extent do leaders show active and visible support for the HKTP?

- How is the leadership supporting the HKTP? What do you wish they are doing?
- To what extent does the PI show active and visible support for the HKTP intervention? (admin only)
- To what extent does the leader indicate his/her communicate the priority of the HKTP implementation?
- To what extent do you/does the PI/admin communicate with staff about HKTP changes?
- To what extent does the PI/admin obtain feedback and input about the HKTP changes?
- To what extent is leader responsive to requests for support to eliminate barriers?
- To what extent are needed resources, access to stakeholders, space requirements, etc. committed and provided in a timely manner?
- What kind of support have senior leadership given you? Do you have enough resources? (PI only)
