# Supplementary material for: Implementation and modification of an organizational-level intervention: a prospective analysis
Source: Implement Sci Commun. 2022 Jun 3;3:59. doi: 10.1186/s43058-022-00296-0 (PMC9164326; doi:10.1186/s43058-022-00296-0)
Supplement: Supplementary file 3 — Additional file 3. Modification Description, Reason, Illustrative Quotation, Modification Type, and Initiator by Study Site. This file presents a description of modifications, the reasons why they occurred, modification type, and illustrative quotations by study site. [file 43058_2022_296_MOESM3_ESM.docx]

| **Additional file 3. Modification Description, Reason, Illustrative Quotation, Modification Type, and Initiator by Study Site** | | | | | |
| --- | --- | --- | --- | --- | --- |
| **Site** | **Modification** | **Reason** | **Illustrative Quotations** | **Type** | **Initiator** |
| **Site-A** |  |  |  |  |  |
| 1A | There was no bilingual/bicultural (Spanish /English) outreach staff to perform lobby days at dialysis centers. There were delays in finding an outreach staff. Although one bilingual staff was hired, he was soon let go, and delays in hiring a replacement resumed. Thus, an English-speaking SW/RN, delivered outreach to dialysis staff about the HKTP  until the bilingual outreach staff was hired. | Goal: NA | “Dr. [T], I think you know, and so we had hired a person initially, who worked with us for a couple months, that did not work out. Since then, we’ve had a position posted for essentially 9 months, and while we have had many, many applicants, none, we’ve interviewed several people, and it has not been a good match. Usually not, usually it’s just once they find out what they position is, they want more direct patient care, something like that. But we do have another interview tomorrow, and the challenge has been, amazingly, finding bilingual nurse or social worker. We’ve got it posted two ways, we have two different recruiters who are actively working with us. We’ve posted it on the UNOS and NATCO website, we’re trying to be proactive about it, but it has definitely been a challenge. And so [Ms. X], who is here with us today too, is our outreach coordinator, and she’s absolutely promoting this, and [the bicultural/bilingual research coordinator] is helping some. But we still don’t have this full time dedicated person.“ (2009) | Substitution | Team* |
| 2A | The bilingual/bicultural study research coordinator accompanied the English-speaking outreach staff to perform lobby days at dialysis centers that have a large Hispanic population.  The research coordinator served as an interpreter to help outreach staff communicate with patients in Spanish. | Goal: To address cultural factors | “And, like I said, the staff are accepting of it and the patients I visit with [our research coordinator] has been wonderful, because I don’t speak Spanish. If I’m going to a clinic that I know is heavily Hispanic population, she’s been able to go with me, and the patients just respond very well to her. And then, if she’s not able, I’m able to get her on the phone as well, I did yesterday, where she talked with them and answered their questions. And that’s been very well received..." (2013) | Substitution | Team |
| 3A | The bilingual/bicultural research coordinator helps translate forms that patients need to fill out at the HKTP clinics because the forms are not yet available in Spanish. | Goal: To address cultural factors | “Yes, and I asked, like they, when I was consenting a patient, and his daughter was there, they’re like, ‘Hey,’ they came to me. They’re like, ‘Hey, can you help us with these papers? They told us to do this.’ I’m like, ‘They don’t have that in Spanish?’ And I asked the receptionist that works at [community nephrology organization] and she’s like, ‘No, we don’t have that yet.’” (2019) | Substitution | Individual |
| 4A | The HKTP brochure in Spanish is not available yet. | Goal: NA | Field notes: Patient brochure: waiting for some minor changes. (operations meeting) | Skipping/ Delay | Team |
| 5A | The institution encountered delays in translating the HKTP information into Spanish on the institutional website. Delays were related to the institution merging with another healthcare institution. Other information about transplantation was previously translated into Spanish on the website. | Goal: NA | Respondent 1: "So, when it comes to the platform, one website, it’s just a general [Site-A] one, that’s not in Spanish at the moment. We still have our central and our north [U.S. state]. And for the north [Site-A], now that we have access to it, I did update to reflect the English content and the Spanish content...  Respondent 2: We have a lot of other information on our website in Spanish around, you know, living donor transplant, frequently asked questions, and things that have always been there in English and Spanish. And so, we hope that, again, it complements this information, that if they want to know more, that is also available in English and Spanish.” (2020 & 2009)  Respondent 1: “Nothing in there is in Spanish on that site. Just –  Respondent 2: But it will be? Is your impression that it will be at some point?  Respondent 1: We’re deciding whether it’s gonna be a new site or a smaller version of it.  Respondent 2: Got it. Ok. Ok. But those are marketing high level decisions and aren’t just transplant. They’re a strategy for the healthcare system as a whole.” (2020 & 2009)  Respondent 2: So, one barrier was that we were changing the website format, right? And so that was a delay. ….  EJG:   Could you say a little bit more about…?  Respondent 2: I’ll let you know the most about that.  Respondent 3: Well, we were right in the midst of doing an entirely new platform for [Site-A] overall.  Respondent 2: [Site-A]  Respondent 3: So we have, what we’re now calling it the “old site,” which was [Site-A].com, and the new site, which encompasses both programs of north [U.S. state that Site-A is located in], and our merger with central [U.S. state that Site-A is located in].  Respondent 2: So, about five years ago, [Site-A merging site 1] merged with [Site-A merging site 2]. So, today, we’re [Site A]. And so, we differentiate that we’re north [of] what is in [city 3]. They have a kidney/panc(reas) program there is central [U.S. state]. And so, it’s, like, very Game of Thrones – north, central. And so, we had our [Site-A merging site] website, they had their website. And so, what, with this new platform, it’s pulling everything together. So, it, you know, the idea is that if you go to the [Site-A] website, you would actually see three kidney transplant programs, not the two that were in north. And so, it’s, you know, [city 1, 2, 3]. And so, it’s been a lot of, it’s a huge amount of work to merge those websites where there are, you know, similar services. And so, that’s. Does that kind of? [interjects –yes] That’s a lot of what that is.  Respondent 3: [Unclear] caused a delay. And so, on the old site there was frozen for a very long time. So, we couldn’t make any changes to the old site. The new site wasn’t ready, so we were kind of in that [unclear].  Respondent 1**:** Now we’re on both." (2009, 2020, & 2025) | Skipping/ Delay | Institution |
| 6A | Patient letters and forms are not yet translated into Spanish at [community nephrology organization]. | Goal: NA | “Some of our barriers, which we’re able to, some if it’s pretty easy fixes, we still have some communication in the forms of letters that were- haven’t translated yet. So we’re working on those issues, as we speak. ... we have a few letters that aren’t translated into Spanish at this point. The way the process, application flows, we have to adjust just a little bit, but we’ve got those, we’re working on those.“ (2004) | Skipping/ Delay | Institution |
| 7A | Letters to nephrologists have not been sent out. | Goal: NA | Field notes: Letters not sent yet (in process of going out). (operations meeting) | Skipping/ Delay | Team |
| 8A | Changes were being made to the scheduler’s script. | Goal: Improve effectiveness | Field Notes:   - Schedulers got approval for revised script and sent yesterday - Change to scheduler’s script | Tweaking | Team |
| 9A | Site PI added new content to the HKTP educational PowerPoint slides. Additions included: adding information to show the family how to fill out the online Breeze [medical health questionnaire], and adding information about the transplant surgeons and their years of experience. | Goal: Improve effectiveness | “I know [the administrator] is speaking to the person who created that to see about getting that in Spanish, hopefully sooner rather than later. In the interim, I have kind of changed my class to where some of my last few slides are actually screenshots of the actual questionnaire, so that it doesn’t look terribly foreign to them when they or their potential donors log on. So I’ve been doing that, and so, anyways. I think for us those are the major things, the Breeze application or an application not in Spanish, getting more family members to come to these clinics, those for me are kind of really the major deal breakers, I think, that could make our clinic much more successful. ... I just wanted this to be, even though it’s a HKTP program, I wanted it to be more institutionalized, you know, to [my institution]...” (2006) | Tweaking | Individual |
| 10A | Site PI did not address cultural beliefs in the HKTP educational presentation. | Goal: NA | Field Notes:  Cultural beliefs not addressed, except the point of stop listening to gossip, but then a slide on donating. | Skipping/  Delay | Individual |
| 11A | The transplant team adjusted the time of the day, and the days of the week in which the HKTP clinics were held due to  scheduling conflicts with other healthcare providers who needed to evaluate patients on the same day. | Goal: Improve feasibility | “I think it’s going pretty well, I do. And I know that it’s kind of, trying to figure out which day would work best and that kind of thing, but I feel, I really believe we’ve obtained some good quality time. We’ve got the first Tuesday, and then the third Monday now. We tried the first Tuesday, third Wednesday, and we shifted that to a Monday, just for scheduling purposes for Dr. [T] and some of the staff I had.“ (2004) | Tweaking | Team |
| 12A | The HKTP clinic was cancelled because of patient no-shows (insurance, sickness), no patients were scheduled, and/or physicians were not available. | Goal: NA | Field Notes:  Variability: Didn’t have 1 clinic in January because of insurance.  - No clinic next week- no patients scheduled.  - 1st week in August clinic got cancelled – no patients scheduled and MDs on vacation. | Skipping/  Delay | Team |
| 13A | HKTP clinic days require all initial evaluation appointments to be completed in 1 day, but some patient appointments have been split into two different days due to scheduling conflicts with other healthcare providers. | Goal: NA | “... But overall, I feel like it’s gone very well. We’ve been able to incorporate all the appointments as best we can. There have been the occasion, where we’ve have to split the patients‘ appointments on two different days, but that’s not the typical flow." (2004)  “I mean, there’s been, you know, some concerns as far as the scheduling, you know. Like I said, some of the dieticians kind of, [unclear 6:15] but that’s- we do that anyway for pre-transplant. There’s been many times when we have to kind of change appointments around for people when they come in. So, I don’t see that being really a big issue. But besides that, everybody has been pretty supportive.” (2022) | Spreading | Team |
| 14A | There is no patient data system in place that tracks HKTP patient data. | Goal: NA | “Yes, and I think the short answer is then is ‘no,’ we really don’t have a system in place that tracks that information.  Nothing is different than what we do for all of our other patients. ... But I would suspect there’s definitely the ability to do that, because there is an entire data team that I know we work with, that if we need certain, if we would like certain information back from the transplant population we can get those numbers back, usually.” (2017) | Skipping/  Delay | Team |
| 15A | There are no transplant phone lines at [community nephrology organization] for Spanish speakers. | Goal: NA | Field Notes:  No Spanish speaking-N/A. Hospital system- N/A; Transplant Phone Line- 2 separate lines for [community nephrology program] and donors.  Main person who answers speaks English and “notices” that person speaks Spanish transfers to Spanish speaker. No bilingual staff answering. | Skipping/  Delay | Institution |
| 16A | Transplant staff provide a laptop during HKTP clinics to help potential living donors with filling out the English Breeze if they wish to start evaluation at that time. | Goal: Improve fit with recipients | “And for us, for the [Site-A] site, we have a laptop that’s available if the patient, the potential donor has time to sit and start that. We have not had anybody do that yet, but we do have the availability of the computer for them to have help here.” (2004) | Adding | Team |
| 17A | Transplant team is hosting Town Hall Q&A panel discussions in Spanish to mirror their English panels, and uses the Spanish sessions as an opportunity to promote the HKTP. | Goal: Increase reach | "As you mentioned, we did have a really good panel, had a really good attendance, about a month ago. That’s something I told [administrator] I would like to kind of continue on maybe a quarterly basis, to be able to just have any, anybody, recipients, potential donors come and have any questions about living donor kidney transplantation. You know, kind of plug our program at the same time. So, I think that was a success. And hopefully, again, word of mouth, if that gets out, bringing in more individuals to attend, I think that would be, that would be great.” (2006) | Adding | Team |
| 18A | There is no tracking system to match people attending the HKTP who were contacted by [English outreach staff] during her outreach. | Goal: NA | “But, again, my focus is different. I don’t typically come away with their name, their contact information or any part of this part of the research study. I’m, not to say that if I have a patient who wants to give me that information, I certainly take it and bring it back, and I come back with little notes, and things to follow up on. But it’s not a main focus to get a list of the patients I spoke with. Or if I give them an application, I’ll say, “Would you like to give me your name? And I’ll be happy to try to watch for your application.” But it’s not a list that I take down and bring back with me.” (2013) | Skipping/  Delay | Team |
| **Site-B** |  |  |  |  |  |
| 1B | The bilingual outreach staff performed fewer and shorter lobby days at dialysis centers than expected (e.g., half days instead of full days). | Goal: NA | "Nothing I can think of right now. I mean, I know that the expectation is, I mean, based on how you all lay out the plan, is 2 full 8 hours days per week, but right now we’re doing 2 full-4 hour days per week. So, our target based on your goal would be 8 patients per month, per se, to attend each clinic, so twice a month. But those numbers would just have to vary based on the amount of outreach we’re able to do. That’s just more of expectations. We’re trying to reach those goals.” (1022) | Shortening | Team |
| 2B | The bilingual outreach staff tweaks the schedule for visiting dialysis centers depending on his availability. | Goal: NA | Field Notes:  [Bilingual outreach staff] is going weekly. He may double up if he is not able to go once a week. | Tweaking | Individual |
| 3B | The bilingual outreach staff does not talk about cultural issues during visits to dialysis centers. | Goal: NA | Field Notes:  [Bilingual outreach staff] doesn’t tell patients that they talk about cultural issues during HKTP class. | Skipping/  Delays | Individual |
| 4B | The bilingual outreach staff created flyers to be posted in dialysis centers before his visits to advertise his upcoming lobby days. | Goal: Increase reach | “But I mean, yeah the- he found that, for example, he would just show up for a lobby day and so people would kind of co-incidentally know, but sometimes what patients have told him was that, ‘If I knew you guys were coming, I would have brought more people here. Just to talk to you here.’ So we started one adaptation that we created like a flyer for them to post in advance. So that way people know that he's coming, and to answer any questions, and kind of screen people, because I mean, like for the federal emergency insurance people, like, he can talk till he's blue in the face about the program, but it's not really available to them. So it's sort of. He already starts doing screening already. Right, but I mean, I think that seems to be going well. People have expressed a lot of interest. ” (1006) | Adding | Individual |
| 5B | Bilingual/  bicultural research coordinator does outreach with bilingual outreach staff. | Goal: Increase reach | Field Note:  RC did outreach with [bilingual outreach staff] on Wednesday | Substitution | Team |
| 6B | The English-speaking outreach staff attends outreach with the bilingual outreach staff. | Goal: NA | “I go with [bilingual outreach staff] to the majority and I stay maybe an hour or two, just to see what’s going on. ’Cause [bilingual outreach staff] is still new and still learning, you know. Yes, he can talk about the program, but also insurances and, you know, a little bit about the process, but he is doing a great job. The patients just love him. They feel comfortable...” (1016) | Substitution | team |
| 7B | The Institution has not yet approved the HKTP education materials. | Goal: NA | Field Notes:  Still waiting for [Site-B] approvals for the HKTP education materials. | Skipping/ Delay | Institution |
| 8B-A | The site PI has not reviewed the website. | Goal: NA | “So, I mean, part of it is just that it's my status. So if we wanted to design a good one, and I also wanted it to reflect what- if we were going to do a website, what the website says and to be able to put the website on there so. [EJG: Oh, the URL on there.] Correct. So I didn't want it just to be like an empty brochure, and then no other connection to anything. I mean, if you don't have an online presence it's hard to have anything except a piece of paper so. So now we have a draft kind of the content of the Web site, and then we're going to revise that, and hopefully we'll go live in the next few months. But we need to design that brochure a part of the broader education... ” (1006) | Skipping/ Delay | Individual |
| 8B-B | The institution has been delayed in the publishing of the website in Spanish, because the website first needs to be approved by institutional leadership. | Goal: NA | "We are always getting inundated with extreme priorities, especially in marketing, because we have to be the distributor of, like, the funds that we are given to promote the brand. So yes, and it is actually one of the reasons why .org has not been updated with that content yet, is because we had a really big priority come through January. So we had to deprioritize getting this content up, and focus on the other priority, which has been since taken care of. But, as large as we are, we’re still very nimble, and we still lack resources, and there's always conflicting priorities. I think, the one beneficial thing for this program from a [Site-B] perspective, is that it falls with the transplant, and transplant is the same priority area, and it also falls within kidney transplant, which is a targeted sub-service line. So it has the benefit of having all our resources funds, etc., to be able to help support it. Whereas if it fell within GI or non-priority service, we won't be having this conversation, because we wouldn’t even be here to have this conversation. So, transplant is definitely well resour(ced), more well-resourced than a lot of the other service lines, but we still always lack resources, and it’s always a challenge, but I think this is something we’re all committed to.” | Skipping/ Delay | Institution |
| 9B-A | The site PI has not reviewed and approved the HKTP flyer that the outreach staff can hand out during lobby days at dialysis centers. | Goal: NA | “So I haven't yet reviewed it, but I would say hopefully in the next month we'll have a design of what that flyer's supposed to look like, and then it will be printed and go out with [outreach staff] by December. ” (1006) | Skipping/ Delay | Individual |
| 9B-B | The institution has not made the HKTP flyers in Spanish for disseminating to patients during lobby days at dialysis centers. | Goal: NA | “I mean we have international services that does translation for our patients, and I've ask them before if they would translate information. I think in the past when they are more heavily staffed they were able to, but now they're short staffed, and they're not able to. I think [other campus of Site-B] might have that service, and we do have the means of doing it that way. I'm not sure specifically what the barrier is to as to why we don't have this. I've made requests with Dr. [Y] before, and he had said it's a good idea. There may have been a time where he said he was going to give me some feedback on what should be in it, but it just hasn't been done.” (1022) | Skipping/ Delay | Institution |
| 10B | Binders containing transplant information have not yet been translated into Spanish. Thus, English binders are being used for the time being. | Goal: Improve feasibility | “If they're interested. These are the folders that I would give them, so the English ones and the Spanish one that's got all, it's pretty much the same information as- The folder itself it's not in Spanish, we don't have Spanish folders, unfortunately. ... So it's just the [Site-B] binder. I've seen other ones for transplant that are just in English as well. So we just don't have that, and I don't think [Site-B] right now is willing to put one together in Spanish as of yet, and there's no budget in this grant for such things, I don't think, unfortunately. But we do have a lot of our print material in Spanish, so anything related to kidney transplant, kidney and pancreas. And then we have the UNOS 'What every patient needs to know.' And then just living donor information here, and the Informate, and then other living donor stuff, but this is the only one that has the website on it. but that's in English. Now, unfortunately, we don't have this little postcard in Spanish yet.” (1022) | Substitution | Team |
| 11B | Scheduler scripts are being modified. | Goal: NA | Field Notes:  Script modification was submitted to IRB. | Tweaking | Team |
| 12B  MAJOR | The use of in-person interpreters is causing appointments during HKTP clinic days to run longer. Thus, the HKTP clinic days are running longer than 1 day. | Goal: Improve feasibility | Field Notes:  Issue nephrologist: patient appointments are double-booked.  Multiple patients are scheduled in 1 time slot.  Longer consult because an interpreter is being used.  Appointments got pushed back. | Spreading | Team |
| 13B | The institution lacks enough in-person interpreters, which causes delays during the HKTP clinic day. Thus, a bilingual-bicultural transplant nurse and a bilingual-bicultural research coordinator serve as interpreters. | Goal: To address cultural factors | "Right. Well, I think it’s, I have seen just one that’s come, and he kind of goes from room to room. But I’m not really sure. I know [the research coordinator] sends out the email saying who needs a translator, and the times that they have appointments, but I’ve only seen one person there that helps. I mean, she and I have, at times when we’re running behind, we’ve kind of said, well we can help, but you know, it makes it, we kind of delay the day a little bit.” (1013) | Substitution | Individual |
| 14B | Transplant bilingual-bicultural nurses and hospital staff are helping Spanish-speaking potential living donors with completing the Breeze [medical health questionnaire] online by telephone and in person. | Goal: Improve effectiveness | “I am glad I saw it. I mean, things like that don't usually work that quickly, honestly. I've been in donations for years and it doesn't usually- someone isn't usually providing information, or if they are they've already thought it through. You know, they come forward, they've already thought it through. But the warmth in that room and response to the presenters, and the comfort level, was amazing. I mean this family, I could barely go to the cafeteria without them. Like hello, they would seek me out. And I mean I always turn to them and I actually I mean, all my team members are like this, but I walked them, because they didn't have a computer. I took them to our library and said, “‘You can use this computer.’ The librarian stepped up and said, ‘I'll help them.’ So that was pretty darn cool, you know, to have all that going on. And then I saw them in the cafeteria next to me, they must think all I do is eat <laughs> but I don't. And they were like, ‘I did the form! I did the form!’ So they knew how to get that out there. Yeah. And they weren't the most sophisticated, individuals. But their passion to help this person was there. You know, they needed a little guidance on how to do things.” (1008) | Adding | Individual |
| 15B | The transplant team has made edits to the HKTP education slides because they found that some slides lacked information on the effectiveness of transplantation or the level of detail was excessive for patients, and to make sure that they are culturally targeted. They also added a note on the slides to say that the research coordinator will provide assistance with Breeze [medical health questionnaire for potential living donors]. | Goal: NA | “We have, actually, we have tweaked the slide presentation. So we found that some slides were like kind of not that effective and went into a level of detail that was excessive for that moment. And that lot of that could be addressed later in the process. But, you know, little things like that, yeah, we have tweaked. But the overall, kind of direction of it, I don't think so. ... I mean, I think people are going kind of above and beyond what they currently are asked to do anyway. You know, for example, [bicultural/bilingual nurse] is an inpatient nurse and she is at every clinic, but she does much more than that. She is helping edit the content of the slides to make sure that it really kind of fits from a cultural perspective. You know, she- that's just one example...“ (1006) | Tweaking | Team |
| 16B | The transplant team has not scheduled the target number of patients to attend the HKTP education sessions because of limits imposed by the room size. | Goal: NA | Field Notes:  Scheduling patients for HKTP  - Can only schedule it to 6 patients per HKTP.  - The transplant waiting room is used by 6 specialites: heart, lung, etc., So the lobby is not exclusively for transplant patients only. | Tweaking | Team |
| 17B | The HKTP clinic was cancelled because patients had cancelled due to sickness and complications with dialysis. | Goal: NA | Field Notes:  - Last clinic was cancelled  - Research coordinator had a feeling one person was going to cancel.  - One young girl, complications w/dialysis  - 2nd had scheduling issues/family was not able to attend | Skipping/  Delay | Team |
| 18B | The bilingual-bicultural transplant nurse taught the HKTP education session instead of the bilingual-bicultural transplant physician (Dr. X) because the physician was busy with a surgical case. | Goal: NA | Field Notes:  Dr. [X] had a surgical case that morning, [nurse] taught the class. | Substitution | Team |
| 19B | The partially bilingual site PI attends the HKTP education sessions and answers questions for patients. | Goal: Improve effectiveness | “...So I've observed that. And a lot of times there will be questions that come up that I may be the only person in the room that knows the answers to. And I typically if I can respond in Spanish I do, and if I can't, I respond in English, and then there are multiple people there that can easily kind of convey that information.” (1006) | Adding | Individual |
| 20B | Bilingual-bicultural nurses and non-bilingual-bicultural transplant nurses attended the HKTP education sessions to help answer questions. | Goal: Improve effectiveness | “Ok, you know I'm one of the three donor coordinators. And each-each one of us attend the classes. So we kind of rotate that through the donor section of classes. So when Dr. [X] is presenting his information we're there to coordinate with him, and help answer some questions. Because we're the first line in the care team that takes care of the patients, you know, for the donor, you know, the Hispanic donors, Spanish-speaking donors, whatever the population is. So we attend that. We have been asked to meet with some of the individuals after the classes, periodically, but not in a formal fashion yet. Kind of on the fly. The physicians have asked us to do that." (1008) | Adding | Team |
| 21B | Bilingual-Bicultural transplant nurses help the partially bilingual site PI to conduct the wrap-up sessions. | Goal: To address cultural factors | “So I think it's, like [nurse], for example, she said: ‘You know, I think things are a lot more effective when you do the wrap ups.‘ [EJG: When you do?] Yeah. [EJG: As opposed to her?] No she- we’re there together. [EJG: Oh! OK.] But she said, you know, in the cases where I haven't been there to do the wrap up, some other physician does them. And so, they're just not as engaged in the concept of it.” (1006) | Adding | Team |
| 22B | There is no patient data system in place that tracks HKTP program patient data for quality assurance. | Goal: NA | "No. I mean, I don't think that there's anything being collected except what we're sending. The only way that people even tabulated to be in the HK(TP) like, we're marching in is when they put their clinical record into EDIT, into our transplant database. They’re marked as being a part of the study. But, as far as, we're not really doing anything else.” (1006) | Skipping/ Delay | Team |
| 23B | A tracking system had not yet been established to match people who the bilingual outreach staff had contacted during lobby days to the people attending the HKTP clinic to determine the effectiveness of outreach efforts. | Goal: NA | “I think we just need to have a better tracking of the patients listed, and something that [outreach staff] and I were talking about this week, perhaps creating a list that I can, that we can compare. Because it could take, you know, we mentioned, anywhere from 3 weeks to even 4 weeks to whether or not the patient could decide on coming to [Site-B]. Or talk to the social workers from their dialysis center to whether or not they can refer the patients. [Outreach staff] is actually following up on those patients 2 weeks after the outreach clinic. So it’s something that him and I need work on it a little bit better. Perhaps I may need to review all his sheets and compare with all the patients that have attended our Hispanic Transplant Program. It’s something that I need to do manually which it will take a long time to compare those names.” (1029) | Skipping/  Delay | Team |
| 24B | The transplant team changed the patient referral form to add a question asking if patients prefer the HKTP clinic in Spanish. | Goal: NA | Field Notes:  Changing form–have an option: Would you like a class/clinic in Spanish; Referral form has additional option. | Adding | Team |
| 25B | The bilingual/  bicultural research staff wants to create a folder containing educational materials to give to potential living donors/family members attending the HKTP clinic. The site PI has approved some of the materials. | Goal: Increase reach | Field Notes:  Research coordinator feels that people who go to the class and want to be donors walk out with no information- nothing to read. Research coordinator found some forms. English/Spanish research coordinator wants to create folder specifically for donors; Asked site PI, he said that’s fine. | Adding | Team |
| 26B | The transplant team changed the HKTP clinic day schedule in terms of the order of clinicians for patients to visit. This was done to accommodate nephrologists’ preferences in working with interpreters in person rather than interpreters via iPads. | Goal: Improve feasibility | Field Notes:  Nephrologists are getting overwhelmed w/ back to back interpreter app; Wednesdays nephrologists have to see patients morning and after; 6 patients after education class; 3- 2pm patients can‘t provide 3 interpreters.  New template will be specifically for HKTP days; Will be based on the calendars (4-5 weeks)  - All changed template = schedule  - All Wednesdays- nephrologist have to see patients in morning and after, not part of idea of HKT.  - Changed 4 in am, 4 in PM instead of all in PM.  · Interpreting services in process of hiring 3 more interpreters, 1 hired + interviewing 2 more.  · Nephrologists don’t like the ipad like Skype, but nephrologists like in-person interpreters because we have in-house Spanish interpreters  · Will start in 2018- new schedule. | Reordering | Team |
| 27B | There are no Spanish transplant phone lines. | Goal: NA | “Well, one of the things that is at [Site-B] is, it [the phone line] presents a challenge in each aspect of the things that we talked about... The clinic’s philosophy in general is that we provide the same care at every site no matter what door you are walking into. So that presents a problem in and of itself because even though we have that philosophy, the programs by nature of their geographic footprint are different. So, for example, instituting something like this for people [to] call [Site-B] and have a Spanish speaking person pick up the phone is…. something that is difficult sort of administratively to have that only in the transplant center because it doesn’t, I guess, it’s not representing all the clinical areas and all the clinical spaces…, it’s an issue every time we try to do something and that’s not just this program, it’s every aspect of our program. So, I mean, that’s one of the philosophical things but it ends up being, everything takes 40 meetings to get done, so…” (1006) | Skipping/  Delay | Institution |
| 28B | Transplant staff provide a laptop during HKTP clinics to help potential living donors with filling out the English Breeze. | Goal: Improve fit with recipients | Field notes:  Research coordinator can help potential living donors to complete Breeze on-site- in exam room or in private computer booths or in library (phone call, 10/11/17) | Adding | Team |
| 29B | Scheduler script is split between schedulers and nurses. First, referral nurses reach patients to inform them about the HKTP. Second, schedulers call to schedule patients and continue to inform them about the HKTP. | Goal: NA | Field notes:  - Brought in contract nurse to do all referring phone calls  - None of the RN speakers are Spanish, there is always an interpreter on the calls | Repeating Elements | Institution |
| *Team refers to the transplant team implementing the intervention, RC= Research Coordinator | | | | | |
